# Beneficial Effect of the Mediterranean Diet on the Reduction of Prediabetes—Results of the Bialystok PLUS Study

**DOI:** 10.3390/nu17122034

**Published:** 2025-06-18

**Authors:** Magdalena Zalewska, Małgorzata E. Zujko, Jacek Jamiołkowski, Małgorzata Chlabicz, Magda Łapińska, Karol A. Kamiński

**Affiliations:** 1 Department of Population Medicine and Lifestyle Diseases Prevention, Medical University of Bialystok, Waszyngtona 15b, 15-269 Bialystok, Poland; jacek.jamiolkowski@umb.edu.pl (J.J.); m.chlabicz@op.pl (M.C.); magda.lapinska@umb.edu.pl (M.Ł.); karol.kaminski@umb.edu.pl (K.A.K.); 2 Department of Food Biotechnology, Medical University of Bialystok, Szpitalna 37, 15-295 Bialystok, Poland; malgorzata.zujko@umb.edu.pl; 3 Department of Cardiology and Internal Medicine with Cardiac Intensive Care Unit, Medical University of Bialystok, M. Skłodowskiej-Curie 24A, 15-276 Bialystok, Poland

**Keywords:** population, prediabetes, prevention, nutrition, Mediterranean diet

## Abstract

**Background:** The Mediterranean diet is considered one of the healthiest and safest diets for preventing chronic diseases. The primary objective of this study was to assess the association between adherence to the Mediterranean diet and the occurrence of prediabetes in a representative population of Bialystok, Poland. Prediabetes is a condition characterized by elevated blood glucose levels that are higher than normal but not yet in the diabetic range, indicating an increased risk of developing type 2 diabetes. **Methods:** The study participants were selected into healthy control (HC) and prediabetic (PreD) groups based on age and gender. Biochemical measurements included total cholesterol (TC), low-density lipoprotein cholesterol (LDL-C), high-density lipoprotein cholesterol (HDL-C), triglycerides (TG), fasting glucose (FG), glycated hemoglobin (HbA1c), high-sensitivity C-reactive protein (hs-CRP), interleukin-6 (IL-6). Additionally, blood pressure, handgrip strength, anthropometric parameters, and body composition were measured. Information on patients’ social data, medical history, and lifestyle history was collected using questionnaires developed for this study. A standardized questionnaire, the Satisfaction with Life Scale (SWLS), was used to assess life satisfaction. Dietary total antioxidant capacity (DTAC) and dietary total polyphenol intake (DTPI) were determined using a 3-day nutritional interview and appropriate databases containing information on polyphenols and the antioxidant potential of food products. To assess adherence to the Mediterranean diet recommendations, a 9-item Mediterranean Diet Index (MDI) was used. **Results:** It was found that the mean MDI for the entire group was low (3.98 ± 1.74), and the HC was characterized by a significantly higher MDI compared to the PreD. A statistically significant positive correlation was found between MDI and HDL-C, whereas a negative correlation was found between MDI and FG, homeostatic model assessment for insulin resistance (HOMA-IR), diastolic blood pressure (DBP), IL-6, body mass index (BMI), waist-hip ratio (WHR), waist circumference, visceral fat mass, android/gynoid fat ratio. **Conclusions:** Abdominal obesity was shown to significantly reduce life satisfaction. In model 3, after adjusting for age, sex, dietary energy intake, alcohol consumption, and smoking, each additional MDI point indicated a 10% lower risk of prediabetes.

## 1. Introduction

Diabetes is one of the most common chronic diseases in the world, which reduces the quality of life by impairing physical and mental well-being, shortens life expectancy, and increases all-cause and cause-specific mortality risks [1]. According to the International Diabetes Federation (IDF), currently, 10.5% of the world’s adult population suffers from diabetes, and half of them remain undiagnosed. Although diabetes is a multifactorial disease, lifestyle factors such as unhealthy diet, low physical activity, obesity, and smoking play a key role in the development of type 2 diabetes (T2D) [2]. Prediabetes usually develops long before the onset of T2D and can persist for many years. The first-line treatment for prediabetes should be lifestyle modification, which has been shown to be associated with greater benefits than pharmacological treatment [3].

Among the modifiable risk factors, much attention has recently been paid to proper eating habits. The American Diabetes Association (ADA), in the “Standards of Diabetes Care” (2024), emphasized the importance of nutritional therapy for people with diabetes. According to these recommendations, it is important to promote healthy eating patterns rather than focusing on individual nutrients or foods [4]. Several eating patterns, including carbohydrate-restricted, low-fat, vegetarian, or vegan, DASH (Dietary Approaches to Stopping Hypertension), and Mediterranean diets, have shown benefits for diabetes management. Healthy eating patterns should be based on key nutritional principles, including consumption of minimally processed plant foods (vegetables, fruits, legumes, whole grains, nuts, and seeds), low-fat dairy products, and fish while limiting consumption of highly processed products, red meat, sweets, sugar-sweetened beverages, and refined grain products [5]. Such food products are characterized by high antioxidant potential high content of polyphenols, vitamins, minerals, unsaturated fatty acids, and dietary fiber, which play an important role in the prevention of diabetes [6,7].

Clinical and observational studies have shown that higher adherence to the Mediterranean diet is associated with lower levels of inflammatory markers such as IL-6 and CRP, better glycemic control, and a reduced risk of metabolic syndrome and diabetes-related complications [8].

The results of a dose–response meta-analysis based on 16 prospective studies from the USA, Spain, Greece, the UK, Denmark, France, Germany, Italy, the Netherlands, Sweden, Norway, Iran, Australia, and Singapore showed a significant inverse association between greater adherence to the MedDiet and diabetes risk in a total sample of over 750,000 people from various countries. It was found that each 1-point increase in MedDiet score led to a 3% reduction in diabetes risk [9]. An intervention study conducted among US adults with prediabetes or T2D showed favorable improvements in glucose levels and lipid profiles with the MedDiet and the ketogenic diet, but higher nutrient content and ease of use favored the MedDiet in the long term [10]. Moreover, an analysis of clinical trials over the last 15 years has shown that among various diets (MedDiet, low-carbohydrate diet, high-protein, and low-fat diet, plant-based diet, time-restricted eating, intermittent fasting) supporting the treatment of diabetes, MedDiet is the most effective and safest diet, devoid of side effects [11]. The MedDiet is not only a typical meal plan but also a lifestyle that includes physical activity, graphically presented in the MedDiet Pyramid [12]. Some authors indicate that better adherence to the MedDiet is positively associated with health-related quality of life [13,14]. However, a Switzerland study found no significant association between adherence to a Mediterranean diet and the risk of developing T2D in a non-Mediterranean population [15], which may suggest a potential modifying role of local dietary practices.

Previous research has shown that the majority of a representative group of the Polish population is characterized by low diet quality, which may predispose to the development of chronic diseases [16]. The aim of the study was to assess the association between adherence to the principles of the Mediterranean diet and the occurrence of prediabetes in a representative population of Bialystok, Poland. Białystok is a city located in the northeastern agricultural part of Poland, commonly referred to as the Green Lungs of the country. Therefore, the dietary habits of the inhabitants of this region may differ from other regions of Poland. Previous studies have shown that greater adherence to healthy eating patterns is also associated with higher levels of life satisfaction [17]. Therefore, life satisfaction, assessed using the Satisfaction with Life Scale (SWLS), was included as a complementary psychosocial dimension to examine whether healthier dietary patterns may be associated not only with a reduced risk of prediabetes but also with higher levels of life satisfaction.

## 2. Materials and Methods

### 2.1. Ethical Approval

This study was approved by the Ethics Committee of the Medical University of Bialystok (Poland) on 31 March 2016 (consent number: R-I-002/108/2016). All participants gave written informed consent following the Declaration of Helsinki.

### 2.2. Study Population

**Figure 1:**
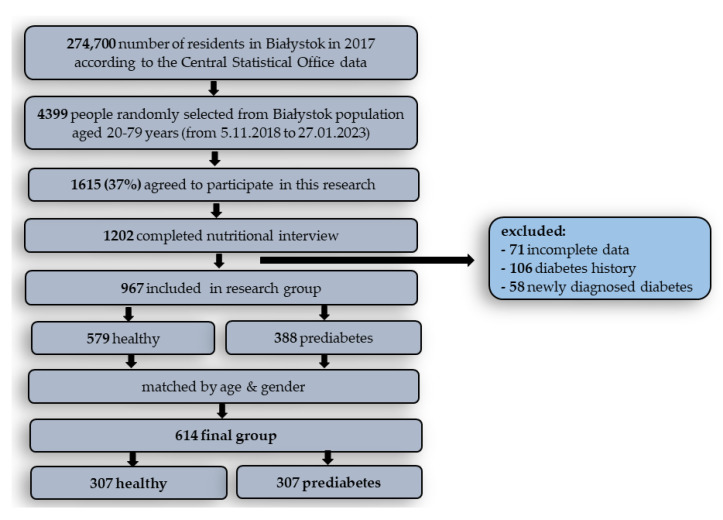
Flowchart of the study population.

This study was conducted as a part of the Bialystok PLUS cohort study, which represents a random sample of Bialystok residents aged 20–79 years. The participants of the study were randomly selected from among the city’s inhabitants in such proportions as to obtain a distribution of proportions similar to the distribution of the city’s population [18]. According to the Central Statistical Office data, the number of residents in Bialystok in 2017 was 274,700. This survey covers the period from 5 November2018 to 27 January 2023. At this time, 4399 people were randomly selected and invited to participate in the study, of which 1615 people (37%) signed informed consent. From this group, 1202 participants completed a nutritional interview. After taking into account the exclusion criteria: incomplete date (71 people), history of diabetes (106 people), and newly diagnosed diabetes (58 people), 967 people were included in the research group. Diabetes was diagnosed if one of the following criteria was met: fasting glucose—FG ≥ 126 mg/dL, oral glucose tolerance test—OGTT ≥ 200 mg/dL, and glycated hemoglobin—HbA1c ≥ 6.5% [19]. There were 579 people without metabolic disorders and 388 people with prediabetes. To compare these groups, we made selections based on age and gender. Finally, 614 people were taken for further analysis, 307 with healthy controls (HC) and 307 with prediabetes (PreD). The flowchart of the study population is shown in Figure 1.

### 2.3. Data Collection

Biochemical measurements in blood were performed at the research center of Bialystok PLUS study and included total cholesterol (TC), LDL cholesterol (LDL-C), HDL-cholesterol (HDL-C), triglycerides (TG), fasting glucose (FG), glycated hemoglobin (HbA1c), high-sensitivity C-reactive protein (hs-CRP), interleukin 6 (IL-6). Oral glucose tolerance test (OGTT) was performed only in subjects without diabetes. Homeostatic model assessment for insulin resistance (HOMA-IR) was calculated from the following formula: HOMA-IR = fasting insulin (mU/L) × fasting glucose (mmol/L)/22.5 [20].

Anthropometric measurements such as height, waist, hip, and thigh circumference were measured using a tape and expressed in cm (SECA 201 tape, Hamburg, Germany). Body mass index (BMI) was calculated as body weight in kilograms divided by height in meters squared and expressed in kg/m^2^. The waist-to-hip ratio (WHR) was calculated as the ratio of waist-to-hip circumference.

Blood pressure (BP) was measured by an automatic Omron M6 Comfort device (Omron Healthcare, Kyoto, Japan) 3 times in the sitting position after 5 min of rest, and an average value was calculated. Handgrip strength was measured using a digital handgrip dynamometer (SAEHAN DHD-1, Saehan Corporation, Masanhoewon-Gu Gyeongsangnamdo, Changwon-si, Republic of Korea). Participants were asked to apply maximum grip force for 3 to 5 s. The procedure was performed 5 times with each hand alternately, with a break of 1 min between each measurement. The average value of the measurements was calculated.

The body composition was measured by dual-energy X-ray absorptiometry (DEXA, GE Healthcare Lunar, Chicago, IL, USA). The average percent fat, visceral fat mass, android fat mass, android lean mass, gynoid fat mass, and gynoid lean mass were measured automatically. Android to total fat ratio and gynoid to total fat ratio were calculated as the ratio of android and gynoid (respectively) fat to total body fat. The android to gynoid fat ratio was calculated as android (central) to gynoid (hip and thigh) fat region [21].

Information on patients’ social data, medical history, and lifestyle history: smoking, physical activity, and diet was collected using questionnaires developed for the Bialystok PLUS study. Participants were asked whether they currently smoked. Response options included: “yes”, “no”, or “I refuse to answer”. Physical activity engagement was assessed separately in summer and winter. The response categories were “every day”, “2–5 times a week,” “once a week”, “less than once a week”, and “not at all”. Missing values on key lifestyle variables (e.g., smoking, physical activity) were accounted for using complete-case analysis to ensure consistency across models.

The diet of the respondents was assessed based on a 3-day nutritional interview. Each participant was asked to record all food, meal, and beverage consumption over a three-day period (two weekdays and one weekend day) using a standardized dietary record form. The size of food portions was estimated using an album with photos of the most frequently consumed products and dishes in Poland [22]. The energy and nutrient values obtained from the diet assessment were calculated using the Diet 6.0 computer program (developed by the National Institute of Public Health, Warsaw, Poland). The dietary assessment included a range of macronutrients (e.g., total protein, animal and plant protein; total fat, saturated fatty acids, monounsaturated fatty acids, polyunsaturated fatty acids, cholesterol; carbohydrates, including starch, sugars, fiber; and alcohol) as well as selected minerals (such as sodium, potassium, calcium, magnesium, and iron) and vitamins. Dietary total antioxidant capacity (DTAC) and dietary total polyphenol intake (DTPI) were calculated based on food consumption data and relevant databases containing information on the polyphenol content and antioxidant potential of foods [23].

### 2.4. Mediterranean Diet Index (MDI)

To assess adherence to the Mediterranean diet recommendations, a 9-item Mediterranean Diet Index (MDI) was used, which included the consumption of the following products: 1—vegetables without potatoes, 2—fruits, 3—nuts, 4—whole grains, 5—fish, 6—red meat and meat products, 7—legumes, 8—alcohol, and 9—monounsaturated to saturated fatty acid ratio. One point was awarded if the consumption of vegetables without potatoes, fruits, nuts, whole grains, fish, legumes, and monounsaturated to saturated fatty acid ratio was equal to or higher than the gender-specific median, and 0 points for intake below the median. Whereas, for consumption of red meat and meat products, 1 point was awarded when intake was below the gender-specific median and 0 when intake was equal to or higher than the median. For alcohol intake, a value of 1 was assigned when consumption was between 10–50 g ethanol per day for men and 5–25 g ethanol per day for women. On this basis, three groups of people were identified: people with a low level of adherence (0–3 points), people with a medium level of adherence (4–6 points) and people with a high level of adherence (7–9 points) [24].

### 2.5. Satisfaction with Life Scale (SWLS)

The SWLS is a 5-item scale designed to measure the satisfaction with one’s life. Participants were asked to rate the extent to which each statement applied to their lives on a 7-point scale, with the following points: 1—completely disagree, 2—disagree, 3—tend to disagree, 4—neither agree nor disagree, 5—tend to agree, 6—agree and 7—completely agree. A sten scale was used for the interpretation, where scores in the range of 1–4 stens (score 5–17) are low, 5–6 stens (score 18–23) are medium, and 7–10 stens (score 24–35) are high. The total score ranged from 5 to 35 points, and a higher score indicated a higher level of life satisfaction [25].

### 2.6. Definition of Prediabetes

The diagnostic criteria for prediabetes were defined according to current recommendations. Prediabetes was diagnosed in individuals who did not previously have diabetes, and at least 1 of the following criteria was met: (1). IPG (impaired plasma glucose)—FPG (fasting plasma glucose) = 100 mg/dL (5.6 mmol/L)-125 mg/dL (6.9 mmol/L); (2). IGT (impaired glucose tolerance)—2-h PG (plasma glucose) during 75-g OGTT (oral glucose tolerance test) = 140 mg/dL (7.8 mmol/L)-199 mg/dL (11.0 mmol/L); (3). HbA1c (glycated hemoglobin) = 5.7–6.4% (39–47 mmol/mol) [19].

### 2.7. Statistic Analysis

We performed one-to-one nearest-neighbor matching on age and sex to create comparable subgroups of individuals with prediabetes and healthy controls. Specifically, we used the MatchIt package version 4.7.2 in R version 4.4.2 [26] to estimate a propensity score via logistic regression, including age and sex as predictors. Matches were selected using the nearest neighbor algorithm, with a caliper of 0.2, to limit excessively large propensity-score differences and ensure close matches. We incorporated exact (or Mahalanobis) matching on these covariates within the caliper and set the matching ratio to 1:1. After matching, we assessed covariate balance by comparing summary statistics and standardized mean differences between groups. The final matched dataset was used for subsequent analyses of outcomes. Comparisons of variables between subgroups were analyzed using a chi-square test for categorical variables or with tests for two proportions with Bonferroni adjustment for multiple comparisons. Spearman’s correlations were used to assess the effect of the Mediterranean diet on biochemical parameters and body composition. Descriptive statistics for continuous variables are presented as means and standard deviations, and categorical variables are reported as proportions. Continued variables were compared between groups using the Mann–Whitney test. To evaluate the independent association between the MDI score and prediabetes risk, logistic regression models were constructed, including only key confounders (age, gender, energy intake, alcohol consumption). We intentionally excluded dietary components and potential mediators (e.g., fiber, antioxidant capacity, obesity markers, inflammation) to avoid collinearity and over-adjustment. IBM SPSS Statistics 27.0 (Armonk, NY, USA) was used for all calculations. The criterion of statistical significance was adopted at the level of *p* < 0.05.

## 3. Results

The basic characteristics of the study population are shown in Table 1. The study participants were selected into healthy control (HC) and prediabetic (PreD) groups based on age and gender. The mean age of the entire analyzed group was 52.02 ± 12.66 years, and 60% were men. There were no statistically significant differences in age groups (20–45 years, 46–60 years, and 61–79 years) between HC and PreD. Furthermore, no statistical differences were observed between groups in terms of education, marital status, and cigarette smoking. In this population, approximately 50% of participants had higher education, 70% were married, and 16% smoked cigarettes. In contrast, a statistically significant difference was found with regard to physical activity. PreD participants were more likely to report a lack of physical activity in winter (*p* = 0.017) and less likely to exercise daily in summer (*p* = 0.003) compared to HC individuals. Additionally, handgrip strength in HC was significantly higher (*p* = 0.041) than in PreD.

Due to the characteristics of the study population division, the PreD group was characterized by higher (*p* < 0.001) glucose levels, glycated hemoglobin, and insulin resistance compared to the HC group. Additionally, PreD had higher levels of triglycerides (*p* = 0.003) and inflammatory markers: Hs-CRP (*p* = 0.001) and IL-6 (*p* = 0.025).

Bialystok residents showed average life satisfaction (22.38 ± 5.14), which was significantly higher (*p* = 0.037) among HC in comparison to PreD.

Anthropometric measurements and body composition assessment were presented in Table 2. It was found that BMI (*p* < 0.001), waist (*p* = 0.003) and hip (*p* = 0.016) circumference, visceral (*p* = 0.003), android (*p* < 0.001) and gynoid (*p* = 0.009) fat mass, % total body fat (*p* = 0.001) were significantly higher in PreD than in HC group.

**Figure 2:**
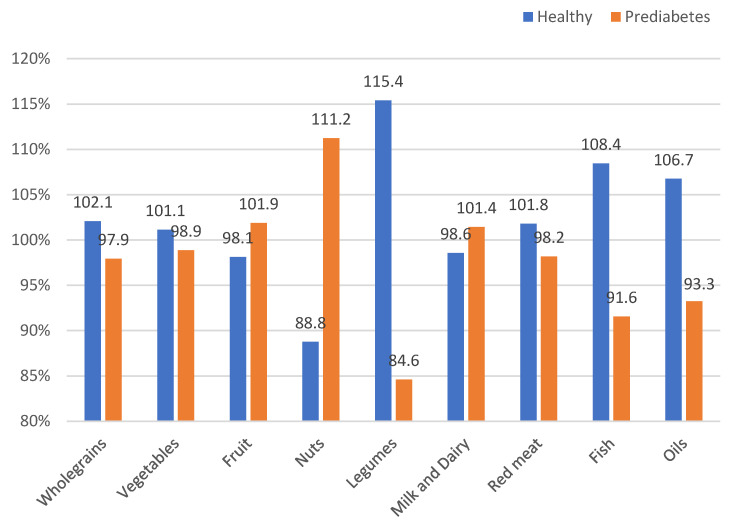
Group average daily intake per body weight (g/kg) in healthy controls (HC) and individuals with prediabetes (PreD) relative to the total sample.

Dietary ingredients and food products in the diet are presented in Table 3. Analysis of mean values of nutrients and dietary products did not show any statistically significant differences between HC individuals and PreD people, except for consumption of *n*-3 fatty acids. The diet of HC contained more (*p* = 0.031) *n*-3 fatty acids compared to the diet of PreD. Moreover, HC consumed more oils, fishes, legumes, and vegetables without potatoes and whole grains but fewer nuts, fruits, milk, and dairy in comparison to PreD, but the differences were not statistically significant. Nut consumption in the entire study group was low and constituted only 30% of the recommended daily intake. The main oil consumed by the study participants was rapeseed oil (90%). Olive oil accounted for only 10% of oil consumption. The average consumption per body weight in each subgroup relative to the average consumption per body weight in the total sample is presented in Figure 2. The healthy group demonstrated higher relative consumption of legumes (115.4%), fish (108.4%), and oils (106.7%) compared to the total group. In contrast, the prediabetes group had a higher relative consumption of nuts (111.2%) relative to the total group. The consumption of milk and dairy products, red meat, whole grains, and vegetables was comparable between the groups.

Adherence to the Mediterranean diet expressed on the 9-point MDI scale is presented in Table 4. The mean MDI for the entire group was low and amounted to 3.98 ± 1.74. The HC group was characterized by a significantly higher (*p* = 0.047) MDI compared to the PreD group. PreD was more likely to demonstrate low MDI than HC (*p* = 0.020), whereas HC were more likely to demonstrate moderate MDI than PreD (*p* = 0.015). Only a few study participants (7.2%) had a high MDI.

**Figure 3:**
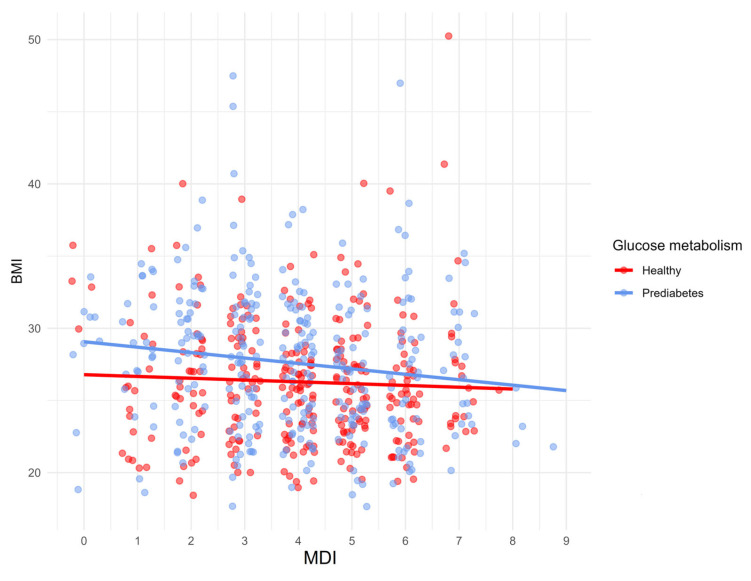
Correlation between MDI and BMI in HC and PreD.

**Figure 4:**
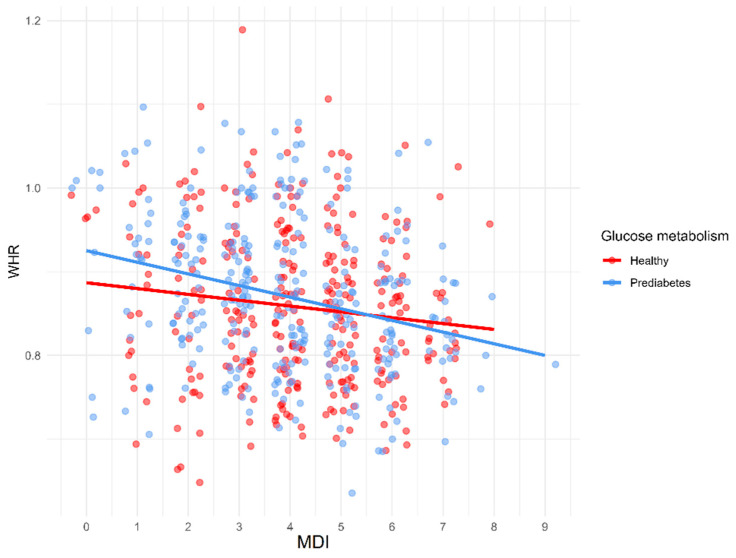
Correlation between MDI and WHR in HC and PreD.

**Figure 5:**
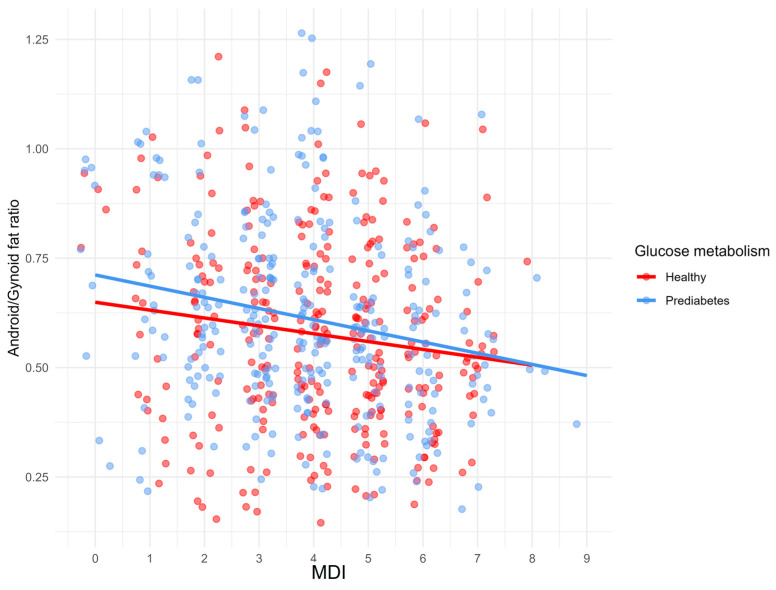
Correlation between MDI and android/gynoid fat ratio in HC and PreD.

The effect of the Mediterranean diet on clinical measurements is shown in Table 5. A statistically significant positive correlation was found between MDI and HDL-C in the whole group (*p* = 0.003) and PreD (*p* = 0.007). Furthermore, a negative correlation was found between MDI and FG (*p* = 0.004), HOMA-IR (*p* = 0.013), DBP (*p* = 0.037), IL-6 (*p* = 0.015), BMI (*p* = 0.001), WHR (*p* < 0.001), waist circumference (*p* < 0.001), visceral fat mass (*p* = 0.030), android/gynoid fat ratio (*p* < 0.001) in total population, between MDI and WHR (*p* = 0.045), waist circumference (*p* = 0.045), and android/gynoid fat ratio (*p* = 0.028) in HC, and between MDI and FG (*p* = 0.025), IL-6 (*p* = 0.006), BMI (*p* = 0.004), WHR (*p* < 0.001), waist circumference (*p* < 0.001), android/gynoid fat ratio (*p* < 0.001) in PreD. Correlations between MDI and BMI, WHR, and android/gynoid fat ratio in HC and PreD are presented in Figure 3, Figure 4 and Figure 5.

A logistic regression model was used to assess the effect of the Mediterranean diet (MDI) on the occurrence of prediabetes. It was found no significant effect in Model 1. After adjusting for age and gender (Model 2), each additional MDI point reduced the risk of prediabetes by 9.3% (*p* = 0.041). In model 3, after adjusting for age, gender, dietary energy intake, and alcohol consumption, each additional MDI point indicated a 10% lower risk of prediabetes (*p* = 0.038) (Table 6).

**Figure 6:**
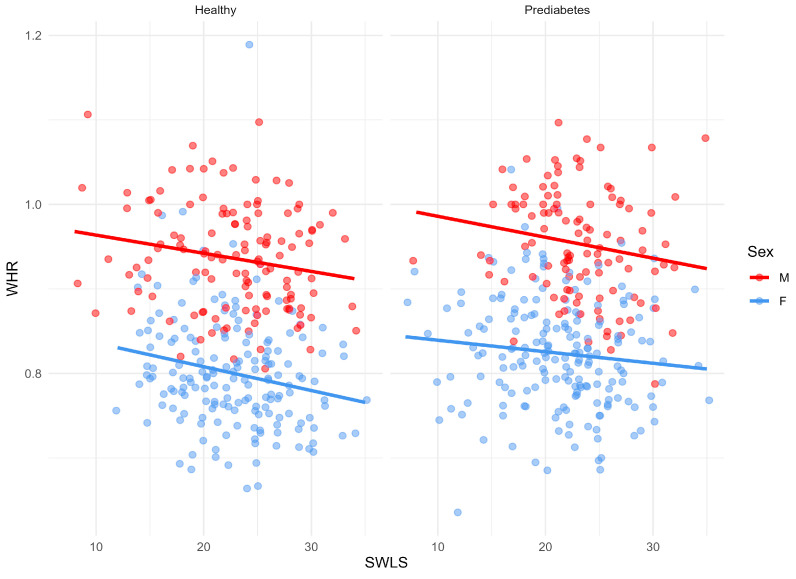
Correlation between WHR and SWLS in men and women in HC and PreD.

Additionally, factors influencing the respondents’ life satisfaction were examined. It was shown that abdominal obesity, expressed by the WHR index, caused a significant decrease in life satisfaction (SWLS) in the population of Bialystok (Figure 6).

## 4. Discussion

Diabetes type 2 is a major public health problem worldwide. In diabetes prevention, it is important to reduce the prediabetes state. Unfortunately, most people with prediabetes remain undiagnosed. Lifestyle changes, including a healthy diet, regular exercise, weight loss, limiting alcohol intake, and quitting smoking, are important first steps in reducing prediabetes and delaying the progression from prediabetes to diabetes [27]. Importantly, such lifestyle changes not only improve metabolic parameters but also enhance overall satisfaction with life, particularly by alleviating symptoms associated with abdominal obesity and chronic low-grade inflammation.

In this study, we analyzed a sample of the Bialystok population selected within the Bialystok PLUS study. Bialystok PLUS is the largest long-term cohort study of the health status of the urban population in Poland. The study (PreD) and control (HC) groups were appropriately matched for age and gender. Furthermore, we found no differences between groups in terms of education level, marital status, and current smoking. However, the PreD group showed significantly lower physical activity and handgrip strength and higher levels of TG, FG, HbA1c, HOMA-IR, Hs-CRP, and IL-6 compared to the HC group. Moreover, the PreD had higher levels of obesity markers, such as BMI, waist circumference, average body fat, visceral fat mass, android and gynoid fat mass. This is consistent with reports from other authors. Obesity is closely related to the development of metabolic disorders, prediabetes, insulin resistance, and inflammation [28,29].

The dietary habits of the PreD and HC groups were not statistically significantly different, except for a higher intake of *n*-3 fatty acids in the HC, which may indicate a beneficial effect of these acids in reducing prediabetes. The HC group also consumed more fish than the PreD group. Some authors have shown that regular consumption of *n*-3 fatty acids from fish ameliorates hyperglycemia in prediabetic individuals by improving muscle glucose transporter 4 (GLUT4) translocation and glucose homeostasis [30].

However, after using the MDI index, significant differences between groups were shown in favor of the HC. Despite the differences obtained, the mean MDI value was low for the entire population of Bialystok and similar to the MDI value in the Dutch [31] and American [32] populations.

To assess adherence to the Mediterranean diet recommendations, we used the 9-point MDI scale. This indicator promotes high consumption of vegetables (except potatoes), fruits, nuts, whole grains, fish, and legumes, as well as low consumption of red meat and meat products and moderate alcohol consumption. In addition, a high ratio of monounsaturated to saturated fats in the diet is preferred [32,33]. Some authors include in the MDI a limited consumption of all dairy products or full-fat dairy products and a high consumption of olive oil [34]. However, limiting the consumption of dairy products is always controversial. Milk and dairy products are an essential part of a balanced diet. They are a source of high-quality protein and easily digestible calcium, and fermented products are a source of lactic acid bacteria. Although full-fat dairy products also contain saturated fatty acids and cholesterol. Results from a large meta-analysis based on 42 publications showed that total dairy consumption is associated with a low risk of obesity, T2D, and hypertension [35]. The inclusion of olive oil in the MDI of the Polish diet is not justified, as the main oil consumed in Poland is rapeseed oil, which is also rich in monounsaturated fatty acids (63% in rapeseed oil vs. 70% in olive oil) [36]. In this study, rapeseed oil accounted for 90% of all oils consumed.

We found a significant positive correlation between MDI and HDL-C in the total population and in PreD and a negative correlation between MDI and FG, HOMA-IR, DBP, IL-6, BMI, WHR, waist circumference, visceral fat mass, android/gynoid fat ratio in total, between MDI and WHR, waist circumference, and android/gynoid fat ratio in HC, and between MDI and FG, IL-6, BMI, WHR, waist circumference, android/gynoid fat ratio in PreD. This indicates a significant effect of the Mediterranean diet in reducing abdominal obesity in HC and glucose levels, obesity, and inflammation in PreD. Our results are consistent with previous reports by other authors [11,37], confirming the beneficial impact of this dietary pattern on cardiometabolic health. Moreover, although the effects of individual dietary components may be subtle, their combined influence may be substantial enough to produce clinically meaningful outcomes [38]. These findings highlight the importance of focusing on overall dietary patterns rather than individual nutrients in chronic disease prevention strategies.

A logistic regression model was used to assess the effect of the Mediterranean diet on the occurrence of prediabetes. It was shown that after adjustment for age, sex, dietary energy intake, alcohol consumption, and smoking (model 3), each additional MDI point resulted in a 10% lower risk of prediabetes in the Bialystok PLUS population. The MedDiet is characterized by high antioxidant capacity, polyphenols, vitamins, minerals, dietary fiber, and monounsaturated fatty acids. The high antioxidant potential of this diet may inhibit oxidative stress, which is involved in the development of insulin resistance and beta cell dysfunction [39]. In this study, total dietary antioxidant capacity was found to be higher in the HC compared to the PreD group, but these differences were not statistically significant. Also, no differences were found in the consumption of polyphenols. Polyphenols exert potent antidiabetic effects by regulating key markers involved in reducing postprandial hyperglycemia and modulating intestinal microbiota [40]. Dietary fiber can slow down the digestion and absorption of glucose and regulate lipid levels [41]. Results of the NHANES population-based study showed that higher intake of mono- and polyunsaturated fatty acids was associated with a decreased incidence of prediabetes and T2D [42]. It is also indicated that MedDiet has a beneficial effect on body weight control, which is the main risk factor for diabetes [43,44]. In our study, abdominal obesity in all participants was associated with lower life satisfaction. Central obesity, in particular, has been linked to increased levels of perceived stress, body image dissatisfaction, and reduced self-esteem, which may contribute to diminished subjective well-being. Other studies also indicate that obesity is associated with poorer health-related quality of life [45,46].

This study has several strengths and limitations. The major strength is that it was conducted on a representative sample of Bialystok residents, and the study and control groups were matched for age and gender. The Bialystok PLUS study protocol was carefully developed and strictly controlled during the study. The main limitation is that the respondents’ diet was assessed based on a three-day nutritional interview that does not reflect habitual and long-term food intake. Moreover, it is very difficult to accurately record all meals, foods, and beverages consumed by study participants over a 3-day period. However, conducting a study on a large, representative group allows us to eliminate some of the errors related to the accuracy of recording the quality and quantity of food consumed. Importantly, due to the cross-sectional design of the study, it is not possible to determine the direction or causality of the observed associations between dietary adherence and the risk of prediabetes or life satisfaction. The results should, therefore, be interpreted as indicative of potential relationships rather than causal effects. To better understand the association between adherence to a Mediterranean diet and the risk of developing prediabetes, including the direction of this association and its potential causal mechanisms, further longitudinal and interventional studies are needed.

## 5. Conclusions

The study found that each additional MDI point meant a 10% lower risk of developing prediabetes in the Bialystok PLUS population. Greater adherence to a Mediterranean dietary pattern can significantly improve metabolic parameters and reduce inflammation and obesity. Given the growing global burden of obesity and metabolic syndrome, such dietary interventions represent an accessible and non-pharmacological strategy for improving metabolic health and reducing the risk of chronic lifestyle-related diseases. Abdominal obesity can result in lower life satisfaction, indicating the broader psychosocial consequences of central adiposity. These findings highlight the importance of early prevention strategies based on lifestyle modification, with particular emphasis on the Mediterranean dietary model.

## Figures and Tables

**Table 1:** Basic characteristics of the studied population.

Variable	Total (N = 614)	Healthy (N = 307)	Prediabetes (N = 307)	*p*
Gender, N (%)				
Men	245 (39.9)	132 (43.0)	113 (36.8)	0.117 ^b^
Women	369 (60.1)	175 (57.0)	194 (63.2)	
Age (years), X ± SD	52.02 ± 12.66	51.36 ± 12.36	52.68 ± 13.18	0.190 ^a^
Age, N (%)				
20–45	206 (33.5)	103 (33.5)	103 (33.6)	
46–60	221 (36.0)	120 (39.1)	101 (32.1)	0.168 ^b^
61–79	187 (30.5)	84 (27.4)	103 (33.6)	
Education ^1^, N (%)				
Below secondary education	90 (14.8)	34 (11.1)	56 (18.3)	
Secondary education	214 (34.8)	101 (32.9)	113 (36.8)	0.068 ^b^
Higher education	309 (50.4)	172 (56.0)	138 (44.9)	
Marital status, N (%)				
Bachelor/Miss	76 (12.4)	33 (10.7)	43 (14.0)	
Married	426 (69.4)	223 (72.6)	203 (66.1)	
Separation/divorced	52 (8.5)	23 (7.5)	29 (9.5)	0.578 ^b^
Widower	41 (6.6)	18 (5.9)	23 (7.5)	
Informal relationship	19 (3.1)	10 (3.3)	9 (2.9)	
Current smoking, N (%)	96 (16.1)	48 (16.2)	48 (15.9)	0.943 ^b^
Physical activity in winter, N (%)				
Every day	62 (10.1)	37 (12.1)	25 (8.1)	0.100 ^c^
2–5/week	122 (19.9)	70 (22.8)	52 (16.9)	0.068 ^c^
1/week	73 (11.9)	36 (11.7)	37 (12.1)	0.899 ^c^
Lack	357 (58.1)	164 (53.4)	193 (62.9)	*0.017* ^c^
Physical activity in summer, N (%)				
Every day	167 (27.2)	100 (32.6)	67 (21.8)	*0.003* ^c^
2–5/week	179 (29.2)	81 (26.4)	99 (32.2)	0.118 ^c^
1/week	43 (7.0)	21 (6.8)	20 (6.5)	0.889 ^c^
Lack	225 (36.6)	105 (34.2)	121 (39.5)	0.187 ^c^
Handgrip strength_max_ (kg), X ± SD	32.95 ± 11.76	33.87 ± 11.86	32.02 ± 11.60	*0.041* ^a^
TC (mg/dL), X ± SD	200.22 ± 40.75	200.66 ± 37.75	199.78 ± 43.59	0.673 ^a^
LDL-C (mg/dL), X ± SD	129.17 ± 36.39	129.12 ± 33.45	129.22 ± 39.17	0.946 ^a^
HDL-C (mg/dL), X ± SD	63.09 ± 17.23	64.15 ± 17.39	62.04 ± 17.02	0.068 ^a^
TG (mg/dL), X ± SD	114.09 ± 70.06	109.66 ± 70.84	118.53 ± 69.09	*0.003* ^a^
FG (mg/dL), X ± SD	100.16 ± 8.49	97.03 ± 6.76	103.29 ± 8.91	*<0.001* ^a^
HbA1c (%), X ± SD	5.43 ± 0.36	5.27 ± 0.24	5.59 ± 0.37	*<0.001* ^a^
HOMA-IR, X ± SD	3.15 ± 2.06	2.74 ± 1.73	3.55 ± 2.25	*<0.001* ^a^
SBP (mm Hg), X ± SD	123.85 ± 16.23	122.73 ± 16.18	124.97 ± 16.26	0.070 ^a^
DBP (mm Hg), X ± SD	81.50 ± 9.96	81.12 ± 9.49	81.88 ± 10.41	0.590 ^a^
Hs-CRP, (mg/L)	1.75 ± 3.34	1.48 ± 2.54	2.02 ± 3.97	*0.001* ^a^
IL-6, (pg/mL)	2.36 ± 5.73	2.26 ± 6.85	2.45 ± 4.44	*0.025* ^a^
SWLS, X ± SD	22.38 ± 5.14	22.77 ± 5.16	21.98 ± 5.10	0.050 ^a^
SWLS, N (%)				
High satisfaction	259 (42.2)	145 (47.2)	114 (37.1)	
Average satisfaction	244 (39.7)	110 (35.8)	134 (43.6)	*0.037 ^b^*
Low satisfaction	111 (18.1)	52 (17.0)	59 (19.3)	

^1^ Education—below secondary (incomplete elementary, elementary school, gymnasium), secondary education (secondary school, postsecondary), higher education (higher school, university); N—number; X—mean; SD—standard deviation; TC—total cholesterol; LDL-C—low-density lipoprotein cholesterol, HDL-C—high-density lipoprotein cholesterol; TG—triglycerides; FG—fasting glucose; HbA1c—glycated hemoglobin; HOMA-IR—Homeostatic model assessment for insulin resistance; SBP—systolic blood pressure; DBP—diastolic blood pressure; Hs-CRP—high-sensitivity C-reactive protein; IL-6—interleukin 6; SWLS—Satisfaction with Life Scale. a—Mann–Whitney test, b—chi^2^ test, c—tests for two proportions with Bonferroni adjustment for multiple comparisons, italic values indicate statistically significant differences (*p* < 0.05).

**Table 2:** Anthropometric measurements and body composition assessment.

Variable	Total (N = 614)	Healthy (N = 307)	Prediabetes (N = 307)	*p ^a^*
BMI (kg/m^2^), X ± SD	26.95 ± 4.69	26.28 ± 4.31	27.62 ± 4.94	*<0.001*
BMI (kg/m2), N (%)				
BMI < 25	232 (37.8)	125 (40.7)	107 (34.9)	
BMI = 25–29.9	237 (38.6)	132 (43.0)	105 (34.2)	*<0.001 ^b^*
BMI ≥ 30	145 (23.6)	50 (16.3)	95 (30.9)	
Waist circumference (cm), X ± SD	87.43 ± 12.40	85.98 ± 12.03	88.88 ± 12.63	*0.003*
Hip circumference (cm), X ± SD	101.07 ± 9.31	100.20 ± 8.77	101.94 ± 9.76	*0.016*
Thigh circumference (cm), X ± SD	58.94 ± 5.86	58.70 ± 5.78	59.17 ± 5.94	0.199
WHR, X ± SD	0.86 ± 0.09	0.85 ± 0.09	0.87 ± 0.09	0.059
Average body fat (%), X ± SD	34.21 ± 0.07	33.23 ± 0.07	35.18 ± 0.07	*0.001*
Visceral fat mass (kg), X ± SD	1.229 ± 0.905	1.122 ± 0.855	1.336 ± 0.941	*0.003*
Android fat mass (kg), X ± SD	2.464 ± 1.189	2.295 ± 1.136	2.632 ± 1.223	*<0.001*
Android lean mass (kg), X ± SD	3.335 ± 0.674	3.352 ± 0.640	3.318 ± 0.707	0.279
Gynoid fat mass (kg), X ± SD	4.166 ± 1.489	4.025 ± 1.455	4.306 ± 1.511	*0.009*
Gynoid lean mass (kg), X ± SD	7.118 ± 1.515	7.175 ± 1.504	7.062 ± 1.525	0.270
Android to total fat ratio, X ± SD	0.089 ± 0.02	0.087 ± 0.02	0.091 ± 0.02	0.053
Gynoid to total fat ratio, X ± SD	0.158 ± 0.02	0.161 ± 0.03	0.156 ± 0.02	0.088
Android/gynoid fat ratio, X ± SD	0.594 ± 0.22	0.576 ± 0.22	0.613 ± 0.22	0.056

BMI—body mass index; WHR—waist-hip ratio; N—number; X—mean; SD—standard deviation. a—Mann-Whitney test, b—chi^2^ test, italic values indicate statistically significant differences (*p* < 0.05).

**Table 3:** Dietary ingredients and food products in diet.

Variable	Total (N = 614)	Healthy (N = 307)	Prediabetes (N = 307)	*p ^a^*
Energy (kcal)	1941.90 ± 614.57	1951.71 ± 618.44	1932.09 ± 611.52	0.592
Protein (g)	83.68 ± 27.17	83.65 ± 27.23	83.71 ± 71	0.918
Carbohydrates (g)	239.80 ± 88.03	241.44 ± 93.59	238.81 ± 88.03	0.838
Monosaccharides (g)	66.01 ± 35.56	66.86 ± 33.89	66.15 ± 37.20	0.750
Dietary fiber (g)	20.39 ± 7.74	20.51 ± 7.82	20.28 ± 7.67	0.794
Fat (g)	74.12 ± 28.33	74.35 ± 27.26	73.89 ± 29.41	0.515
MUFA (g)	30.11 ± 12.39	30.39 ± 12.14	29.83 ± 12.65	0.470
PUFA (g)	12.14 ± 5.73	12.34 ± 5.95	11.94 ± 5.47	0.400
SFA (g)	25.39 ± 11.40	25.21 ± 10.95	25.58 ± 11.84	0.939
MUFA/SFA	0.54 ± 0.31	0.55 ± 0.33	0.52 ± 0.27	0.165
Fatty acids *n*-3 (g)	2.51 ± 1.50	2.59 ± 1.44	2.42 ± 1.54	*0.031*
Fatty acids *n*-6 (g)	9.51 ± 4.83	9.62 ± 5.13	9.40 ± 4.52	0.552
*n*-6/*n*-3	4.39 ± 2.41	4.17 ± 2.03	4.61 ± 2.73	0.054
Magnesium (mg)	332.43 ± 111.74	331.75 ± 108.72	333.12 ± 114.86	0.977
Zinc (mg)	10.79 ± 4.77	10.51 ± 3.28	11.07 ± 5.90	0.339
Calcium (mg)	665.53 ± 297.17	668.31 ± 292.70	662.74 ± 302.02	0.843
Iron (mg)	12.64 ± 8.89	12.34 ± 4.26	12.93 ± 11.83	0.975
Vitamin D (µg)	4.07 ± 3.91	3.98 ± 3.86	4.15 ± 3.96	0.286
Vitamin B_1_ (mg)	1.28 ± 0.62	1.26 ± 0.50	1.30 ± 0.72	0.450
Vitamin B_6_ (mg)	1.90 ± 2.01	1.79 ± 0.77	2.01 ± 2.74	0.555
Vitamin B_12_ (µg)	4.55 ± 3.87	4.56 ± 3.77	4.54 ± 3.97	0.731
Vitamin E (mg)	11.40 ± 5.78	11.54 ± 5.45	11.25 ± 6.09	0.294
Vitamin C (mg)	102.42 ± 148.37	105.47 ± 198.35	99.38 ± 68.82	0.804
DTAC (mmol)	1449.33 ± 667.00	1484.12 ± 675.36	1414.54 ± 657.79	0.083
DTPI (g)	2253.90 ± 782.60	2284.09 ± 778.74	2223.71 ± 786.56	0.270
Vegetables without potatoes (g)	269.57 ± 140.97	271.17 ± 132.46	267.97 ± 149.19	0.406
Potatoes (g)	92.76 ± 70.53	90.19 ± 70.77	95.33 ± 70.21	0.309
Fruit (g)	240.74 ± 202.79	236.08 ± 183.63	245.40 ± 220.50	0.930
Fruit juices (g)	40.06 ± 126.64	35.64 ± 125.30	44.48 ± 133.90	0.081
Nuts (g)	12.71 ± 18.87	11.32 ± 17.86	14.11 ± 19.75	0.109
Legumes (g)	9.56 ± 26.16	11.43 ± 31.09	7.7 ± 19.92	0.178
Wholegrains (g)	52.65 ± 65.42	53.55 ± 66.23	51.75 ± 64.71	0.882
Refined cereal products (g)	162.47 ± 99.44	166.87 ± 111.81	158.06 ± 85.26	0.674
Milk and dairy products (g)	193.66 ± 138.08	189.03 ± 130.88	198.29 ± 144.99	0.500
Eggs (g)	43.62 ± 44.19	42.73 ± 42.68	44.48 ± 45.72	0.966
Meat (g)	146.91 ± 88.53	144.96 ± 87.56	148.87 ± 89.58	0.563
Red meat (g)	103.62 ± 81.54	103.66 ± 83.25	103.57 ± 79.93	0.957
Fishes, g	23.96 ± 36.81	25.91 ± 38.96	22.02 ± 34.48	0.344
Oils (g)	16.13 ± 13.95	17.14 ± 15.36	15.13 ± 12.32	0.096
Olive oil (g)	2.03 ± 6.13	2.22 ± 6.59	1.83 ± 5.63	0.818
Rapeseed oil (g)	14.10 ± 15.24	14.92 ± 16.71	13.30 ± 13.55	0.118
Alcohol (g ethanol)	53.81 ± 180.99	54.33 ± 149.98	53.30 ± 207.68	0.062

Variables were presented as X ± SD; X—mean; SD—standard deviation; MUFA—monounsaturated fatty acids; PUFA—polyunsaturated fatty acids; SFA—saturated fatty acids; DTAC—dietary total antioxidant capacity; DTPI—dietary total polyphenol intake. a—Mann–Whitney test, italic values indicate statistically significant differences (*p* < 0.05).

**Table 4:** Adherence to the Mediterranean diet.

Variable	Total (N = 614)	Healthy (N = 307)	Prediabetes (N = 307)	*p*
MDI, X ± SD	3.98 ± 1.74	4.11 ± 1.66	3.85 ± 1.81	*0.047 ^a^*
Low MDI (0–3), N (%)	238 (38.8)	105 (34.2)	133/43.3	*0.020 ^c^*
Moderate MDI (4–6), N (%)	332 (54.1)	181 (59.0)	151/49.2	*0.015 ^c^*
High MDI (7–9), N (%)	44 (7.2)	21 (6.8)	23/7.5	0.754 ^c^

X—mean; SD—standard deviation; N—number. a—Mann-Whitney test, c—tests for two proportions with Bonferroni adjustment for multiple comparisons, italic values indicate statistically significant differences (*p* < 0.05).

**Table 5:** Correlation between MDI and selected clinical measurements.

Variable	Total (N = 614)		Healthy (N = 307)		Prediabetes (N = 307)	
	R	*p*	R	*p*	R	*p*
TC (mg/dL)	−0.001	0.986	−0.029	0.614	0.027	0.640
LDL-C (mg/dL)	−0.037	0.362	−0.039	0.497	−0.032	0.576
HDL-C (mg/dL)	0.120	0.003	0.074	0.195	0.154	*0.007*
TG (mg/dL)	−0.015	0.706	0.008	0.885	−0.022	0.707
FG (mg/dL)	−0.115	0.004	−0.030	0.597	−0.128	*0.025*
HbA1c (%)	−0.064	0.114	−0.016	0.785	−0.013	0.820
HOMA-IR	−0.107	0.013	−0.049	0.425	−0.103	0.085
SBP (mm Hg)	−0.034	0.399	0.018	0.075	−0.066	0.249
DBP (mm Hg)	−0.084	0.037	−0.088	0.126	−0.069	0.231
Hs-CRP (mg/L)	−0.028	0.488	−0.087	0.127	0.056	0.325
IL-6 (pg/mL)	−0.104	0.015	−0.016	0.799	−0.162	*0.006*
BMI (kg/m^2^)	−0.138	0.001	−0.078	0.174	−0.166	*0.004*
WHR	−0.206	<0.001	−0.114	0.045	−0.285	*<0.001*
Waist circumference (cm)	−0.199	<0.001	−0.115	0.045	−0.261	*<0.001*
Visceral fat mass (kg)	−0.088	0.030	−0.051	0.378	−0.099	0.083
Android/gynoid fat ratio (%)	−0.179	<0.001	−0.126	0.028	−0.211	*<0.001*

TC—total cholesterol; LDL-C—low-density lipoprotein cholesterol, HDL-C—high-density lipoprotein cholesterol; TG—triglycerides; FG—fasting glucose; HbA1c—glycated hemoglobin; HOMA-IR—Homeostatic model assessment for insulin resistance; SBP—systolic blood pressure; DBP—diastolic blood pressure; Hs-CRP—high-sensitivity C-reactive protein; IL-6—interleukin 6, BMI—body mass index; WHR—waist-hip ratio, italic values indicate statistically significant differences (*p* < 0.05).

**Table 6:** Risk of prediabetes in a logistic regression model.

	Model 1.		Model 2.		Model 3.	
	OR	*p*	OR	*p*	OR	*p*
MDI	0.919	0.071	0.907	*0.041*	0.900	*0.038*
Age	-	-	1.007	0.308	1.007	0.290
Sex, M vs. W	-	-	1.340	0.085	1.402	0.073
Energy of diet	-	-	-	-	1.000	0.858
Alcohol consumption	-	-	-	-	0.502	0.264

M—men; W—women; MDI—Mediterranean diet index; OR—odds ratio; Model 1.—unifactorial; Model 2.—adjusted for age and sex; Model 3.—adjusted for age, gender, energy intake, and alcohol consumption, italic values indicate statistically significant differences (*p* < 0.05).

## Data Availability

The original contributions presented in this study are included in the article. Further inquiries can be directed to the corresponding author.
